# The Institutional Library

**Published:** 1919-09-06

**Authors:** 


### THE INSTITUTIONAL LIBRARY.

### The Future of Medicine: as Sir James Mackenzie Sees It.*

At the present moment, according to the authorities of the various medical schools, there is a real glut of new students in medicine—the aftermath of the abnormal conditions of the last five years, as everyone admits. There is, therefore, both an unusual quantity of new blood about to be infused into the profession, and an unusual number of young men, most of them recently emerged from the fiery ordeal of battle, to whom the future developments of medicine are of vital, if not of immediate importance. They may not, from want of first-hand acquaintance with the problems and the tendencies which Sir James Mackenzie in his recently published work describes, be able to weigh the evidence and the probabilities of the future so well as those who have already spent some years of their lives in medical practice; but none the less Sir James's prophecies, his wide outlook and calm sagacity, his philosophic tendencies, as well as his great name and eminent career, combine to demand, even from the youngest and most lighthearted of the "new entry," an attentive hearing.

From this point of view the second of the three parts into which Sir James divides his material may be too technical and recondite to be immediately helpful, though to his professional colleagues it is perhaps the most valuable of the three. To the student it is rather the first and third parts—entitled by their author respectively "critical," and "constructive"—that will most appeal. In the first of these sections he has much to say in criticism of certain methods in medical education which appear to have changed little in the last fifty years, since Sir James Mackenzie was himself a student. One of his first points concerns that much debated question, the formal lecture. He deprecates the plan, feverishly pursued by many students, of hurried and copious note-taking at lectures, with a subsequent attempt to work up these notes into a manuscript which shall replace the text-book. The present writer is one who always preferred text-books to lecture notes, though a good number of his fellows followed the other system which Sir James condemns. Incidentally he says that half-an-hour at a time is quite long enough for most students to listen to a lecture, though he admits that some can concentrate the attention for longer periods.

Another suggestion is that before attending any lecture the student should have some slight acquaintance with the subject to be dealt with, and this requires that the last five minutes of each lecture (in a formal course) should be devoted to giving a brief outline of the subject-matter of the next lecture, so that the students may come prepared "to appreciate" the exposition, "to understand the demonstrations, and to note when the teacher has supplemented the text-book by new material."

After that our author proceeds to a few sly hits at examiners of a certain type, which will probably delight that type of student who sees in examiners mortal and hereditary foes. That there are bad examiners of the kind whose folly he exposes cannot be questioned; happily they are not very common. Probably most teachers will agree with the criticisms of Sir James Mackenzie both on the bad examiners themselves and on the (lack of) system which does not speedily eliminate them from the examination rooms.

He proceeds to a criticism of the total absence from the training-schools of general practitioners. Medical education, as he points out, is conducted entirely by specialists, who know little—and that not by first-hand experience—of the peculiar requirements of general practice, in which by far the great majority of their pupils are destined to engage. In pleading for the addition of experienced general practitioners to the staff of every training-school, Sir James is proposing what would certainly be most desirable if men of the right type (admittedly rare) could be found to take it up: a combination which it is to be feared would be rare. He will perhaps carry more general agreement in his strictures on the excessive proportion of the students' available time spent upon non-essentials, as compared with that allotted to clinical studies. This is an old-standing complaint against modern medical education, and one that has much real substance in it. Far too many precious months are wasted in grounding the student in biology, physics, chemistry, and anatomy. Not that these substances are not in themselves desirable of assimilation: the deeper the student can go with them—consistently with a thorough clinical training—the better equipped he will be. But as matters stand he is taught far too much of them; and in the case of anatomy, he is taught the wrong sort of anatomy. Most of the governing body of the College of Surgeons deny this; they are prone to assert that no surgeon can know too much anatomy, and that more rather than less anatomy should be taught. In this view the present writer cordially disagrees with them, and to find no less an authority than Sir James Mackenzie preaching from the same text affords him considerable gratification. There is much more in this book which will give food for careful thought to all who care for the future of medicine and of the medical profession. If the very brief account here given of only one part of Sir James's analysis and forecast induces students or doctors to study the original for themselves, it is certain that nothing but profit to them will accrue. From those who remember that Sir James Mackenzie was himself a general practitioner in Burnley for eight and twenty years, his views will receive all the more attention because of his unique knowledge of all sides of medical practice and education in this country.

* *The Future of Medicine.* By Sir James Mackenzie. Frowde, and Hodder and Stoughton. 1919. Price 5s. 6d. net.

### The Institutional Library—(*continued*).

**Eugenics and Environment.** By Prof. C. LLOYD MORGAN, LL.D., D.Sc., F.R.S. (John Bale, Sons and Danielsson, Ltd. Price 2s. net.)

THE seven condensed chapters of this book are less a general study of environment in relation to eugenics than an explanation of the means whereby we may study their relation accurately. The methods of application are illustrated by charts and diagrams. Such simple characters as that of height are taken, and by relating height in inches to the numbers of individuals of a given height in some selected group, the normal curve of probability is obtained. With the confidence inspired by the method in attacking a simple problem, the reader is invited to apply it to such physical characters as can be measured, and to such mental qualities as can be estimated. From this we learn that eugenics must cultivate extremely exact methods because the facts which it seeks to study are very difficult to obtain. Being a social science, it desires to estimate tendencies. These are difficult to define accurately. Because it is rather a series of generations than of groups which has to be studied, and because the remedies suggested by the determination of tendencies raise ethical questions, eugenics has perhaps more difficulties than other sciences. Its data are elusive. Hence its methods must be reliable, if its conclusions are to have authority. The study either must be taken seriously or is better left alone, and in making this plain to the attentive reader Professor Lloyd Morgan has enforced a salutary lesson. For this reason we commend the book, since nothing could be more disastrous for the new science than to become a fashionable quarry for glib generalisations or hasty laws.

**Food and the Public Health.** By WILLIAM G. SAVAGE, B.Sc., M.D., D.P.H. (London: Cassell & Co., Ltd. 1919. Pp. 155+vii. Price 5s. net.)

THIS is the first of a series of handbooks dealing with various aspects of public health and to be issued under the general editorship of Sir Malcolm Morris. In a day in which we hear so much of our national defects and delinquencies it is well that the public should have an opportunity of learning of some of our achievements in a department of medical enterprise where we can honestly claim both an early and a prominent place, and the editor of this new series is abundantly well equipped for the task he has undertaken. On patriotic not less than on professional grounds we cordially wish him a full measure of success. The inauguration of a Ministry of Health must inevitably mean a quickened interest in public-health questions and an extension of the numbers of those concerned with the administrative regulation of these questions. There will therefore be a demand for information and guidance on the part of many who are unable to appreciate the technical language of the more severe text-books, and it is to satisfy this position that the new series is intended. The plan carries its own commendation.

The volume on "Food and the Public Health," by Dr. Savage, shows a close recognition of the spirit which we are told is to animate the series. It is a plain and practical statement of the relation of food to health, and while it touches such controversial issues as total abstinence and vegetarianism, it keeps these in due subordination to the larger discussion. The professional reader will hardly find it anything either new or startling, and in certain topics at all events he will judge it to be somewhat scanty. But for the layman cultivating an intelligent interest or administrative efficiency the book is well adapted. It is neither too long nor too learned, and we feel sure it will receive a wide recognition.

**The Problem of Sex Diseases.** By A. CORBETT-SMITH. (Bale, Sons and Danielsson. Second Edition. 1919. Pp. 107. Price 2s. 6d. net.)

SINCE Major Corbett-Smith published the first edition of this booklet in 1914 the whole of the great war has intervened, bringing with it an increase not only of the urgency of the national health, but of the risk to it which venereal diseases constitute. Since 1914 the author has been through the retreat from Mons, of which he has become the most popular chronicler, and has seen the undoubted relaxation of sexual morality which the war has brought in its train. Though not a medical man, he has studied the best medical authors to such good purpose that for a detached view of the evils of venereal disease, apart from actually treating them clinically, he is probably better qualified to obtain a true perspective than most practising medical men. Though here and there a turn of expression may be found which could, in our opinion, be improved upon, yet, broadly speaking, it is a pleasure to acknowledge the tact, the sincerity, the broad-mindedness, and the truthfulness of the author. We commend his exposition very strongly to parents, to the clergy, and to school masters and mistresses. Major Corbett-Smith holds that ignorance and prudishness have been too rife in Britain on this topic, and that both must be dispelled in the interests of the nation at large: certainly he is doing his part manfully in the campaign against these foes, and we wish him all success in it.

**The Medical Register for 1919.** (Published for the General Medical Council by Constable and Co., Ltd., 10 Orange Street, Leicester Square, W.C. 2. Pp. 1212. Price 10s. 6d.)

THIS annual is produced in its usual form. The law as it particularly affects medical practitioners and dentists is summarised in the pages preceding the directory, and is in so convenient a form that, to those who do not need the current year's issue of the Register, the copies of past years, which can be obtained for 2s., should be a good purchase.

**The Dentists Register for 1919.** (Published for the General Medical Council by Constable and Co., Ltd., 10 Orange Street, Leicester Square, W.C. 2. Pp. 140. Price 3s. 4d. Copies for previous years 1s.)

THERE are 5,567 registered dentists in the United Kingdom, the total number appearing upon the Register for 1913 was 5,140.

**Rhymes of the Red Triangle.** Verses by HAMPDEN GORDON, pictures by JOYCE DENNYS. (London: John Lane; New York: John Lane Company; Toronto: J. M. Dent and Sons, Limited. 4s. 6d. net.)

IN verse and coloured picture the author and artist respectively give a bright and amusing indication of the war work of the Y.M.C.A. thus, with novelty of style and expression, set forth:

"What is it that can spread its limbs to reach
From Euston Square to fabulous Baghdad:
That has a thousand arms, and lends with each
A helping hand to cheer the fighting lad:
That follows fast where Freedom's forces go,
Through dust-storms of the Desert, Afric rains,
The mud of Flanders, Macedonian snow,
The palpitating heat of Indian plains;
Whose growth keeps pace with ev'ry changing need
And flourishes the most where battle rages?

. . . . . . . . .

The answer, if you'll stop, and look, and read,
Lies (somewhat camouflaged) within these pages."

The "Movies," the Night Patrols, the Letters Home, the Trench Libraries, the catering and washing-up, and other joys which the Y.M.C.A. provide, are admirably dealt with in this bright little book, which many of those the Y.M.C.A. has aided will welcome and keep as a war souvenir.

