# Erratum

**Published:** 1919-09-20

**Authors:** 


### Erratum.

The publishers (Messrs. Henry Frowde and Hodder and Stoughton) of Sir James Mackenzie's recent book entitled "The Future of Medicine," reviewed in The Hospital of September 6, 1919, point out that the price of this book was there stated to be 5s. 6d., whereas the actual price is 8s. 6d.

